# Anti-inflammatory effects in a mouse osteoarthritis model of a mixture of glucosamine and chitooligosaccharides produced by bi-enzyme single-step hydrolysis

**DOI:** 10.1038/s41598-018-24050-6

**Published:** 2018-04-04

**Authors:** Yali Li, Liang Chen, Yangyang Liu, Yong Zhang, Yunxiang Liang, Yuxia Mei

**Affiliations:** 1 State Key Laboratory of Agricultural Microbiology, College of Life Science and Technology, Huazhong Agricultural University, Wuhan, 430070 P. R. China; 2 Department of Orthopedics, Renmin Hospital of Wuhan University, 9 Zhangzhidong Street, Wuhan, 430060 P. R. China; 3 Hubei Collaborative Innovation Center for Industrial Fermentation, Wuhan, 430070 P. R. China

## Abstract

We developed a novel technique of bi-enzyme single-step hydrolysis, using recombinant chitosanase (McChoA) and exo-β-D-glucosaminidase (AorCsxA) constructed previously in our lab, to degrade chitosan. The hydrolysis product was shown by HPLC, FTIR, and chemical analyses to be a mixture (termed “GC”) composed primarily of glucosamine (80.00%) and chitooligosaccharides (9.80%). We performed experiments with a mouse osteoarthritis (OA) model to evaluate the anti-inflammatory effects of GC against OA. The three “GC groups” (which underwent knee joint damage followed by oral administration of GC at concentrations 40, 80, and 160 mg/kg·bw·d for 15 days) showed significantly downregulated serum expression of pre-inflammatory cytokines (IL-1β, IL-6, TNF-α), and significant, dose-dependent enhancement of anti-inflammatory cytokine IL-2, in comparison with Model group. Levels of C-reactive protein, which typically rise in response to inflammatory processes, were significantly lower in the GC groups than in Model group. Thymus index and levels of immunoglobulins (IgG, IgA, IgM) were higher in the GC groups. Knee joint swelling was relieved and typical OA symptoms were partially ameliorated in the GC-treated groups. Our findings indicate that GC has strong anti-inflammatory effects and potential as a therapeutic agent against OA and other inflammatory diseases.

## Introduction

Chitin, the second most abundant carbohydrate polymer (after cellulose), is widespread in organisms, *e.g*., fungal cell walls, crustacean shells, insect exoskeletons, mollusk radulae and beaks, and fish scales. Chitosan is derived from chitin by deacetylation. To improve the water solubility and bioavailability of chitosan, it is often degraded into chitooligosaccharides (ChOS) or glucosamine (GlcN) by chemical or enzymatic methods. A variety of bioactivities of ChOS and GlcN have been demonstrated, including antitumor^1^, antimicrobial^2,3^, immunomodulatory^4,5^, antioxidant^6^, and anti-inflammatory^7,8^. ChOS and GlcN have been widely applied in the food and pharmaceutical industries, and others.

Osteoarthritis (OA) is a complex disease process that affects entire synovial joints^9^. The etiology of OA may include defective articular cartilage structure, inadequate cartilage biosynthesis, joint trauma, joint instability, and inflammatory mechanisms^10^. Non-Steroidal Anti-Inflammatory Drugs (NSAIDs), the most widely prescribed medications for treatment or rescue analgesia of OA, unfortunately have a high incidence of gastric, renal, and hepatic side effects^11^. Symptomatic Slow-Acting Drugs in OA (SYSADOAs) are intended to relieve OA symptoms without major side effects. GlcN and ChOS can be used as SYSADOAs because of their anti-inflammatory effect, reducing the need for NSAIDs^11,12^. Orally administered GlcN has been used worldwide for effective OA therapy, typically in combination with chondroitin sulfate or other compounds.

Various aspects of GlcN usage remain confusing or controversial. Differing molecular forms of GlcN are present in related products, including generics, over-the-counter (OTC) products, nutritional supplements, and foods. GlcN sulfate (“GS”) has been claimed to be superior to GlcN hydrochloride (“GH”) in some reports^13^, or to provide an oral “boost” to chondroitin sulfate synthesis. There have been conflicting claims regarding efficacy, heterogeneity, and production cost of various products^10,14^. The only organic component in either “GS” or “GH” is the free amino monosaccharide GlcN (C_6_H_13_NO_5_), which should be considered the active ingredient, and may increase the glycosaminoglycan (GAG) precursor^15^.

The differing formulations of GlcN and the resulting controversy arise from the differing production methods. GlcN salts are generally produced by acid hydrolysis in industry, and a high degree of heterogeneity is considered to exist in industry-sponsored trials of GlcN salts^14^. Free GlcN can be prepared by enzymatic hydrolysis. The efficacy in OA therapy of this form of GlcN is ambiguous. Chitosan and ChOS have been shown to enhance bioavailability of GlcN^16,17^. From previous studies, it could be deduced that a combination of free GlcN and ChOS (or chitosan) may be effective for OA treatment.

To test this possibility, we prepared a mixture of GlcN and ChOS (termed “GC”) by bi-enzyme single-step hydrolysis, administered it orally in an OA mouse model, and assessed its *in vivo* anti-inflammatory effects. Our findings will be useful for development of novel anti-inflammatory agents in the nutraceutical industry, and of “functional foods” for prevention or alleviation of OA or other inflammatory diseases.

## Results

### Characterization of GC sample

**Figure 1:**
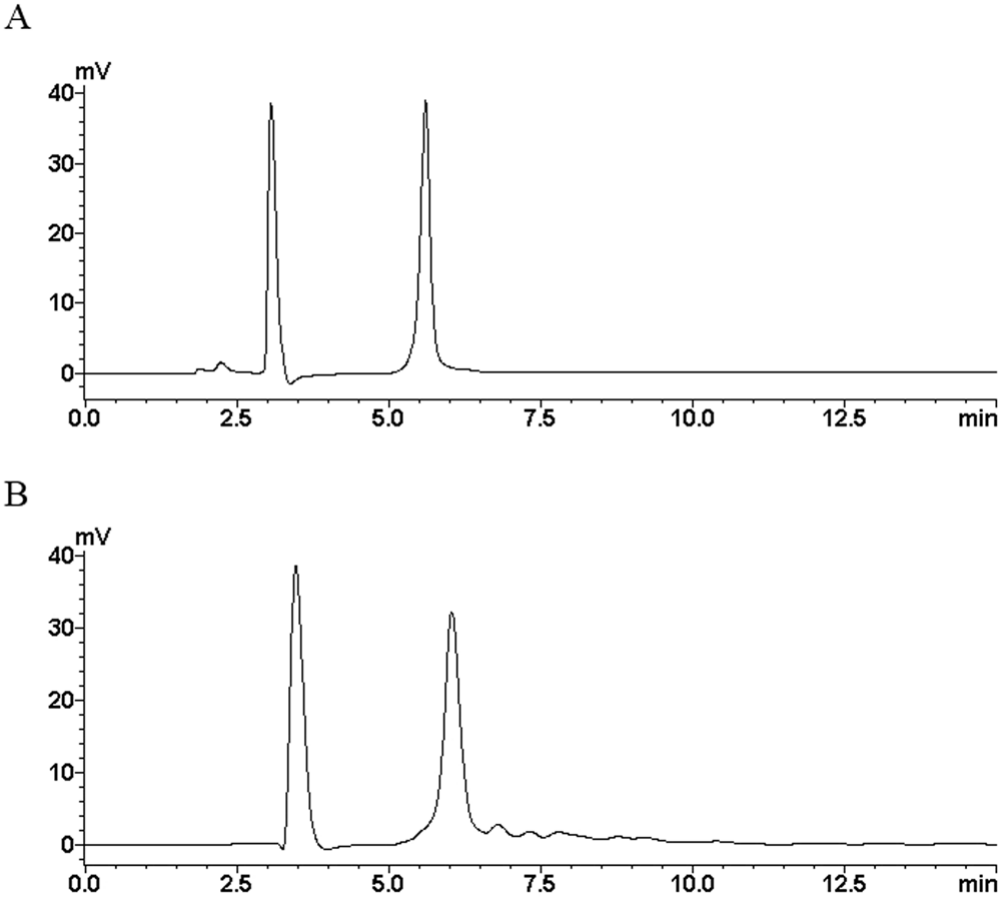
HPLC spectra of standard GlcN hydrochloride (**A**) and GC (**B**).

**Figure 2:**
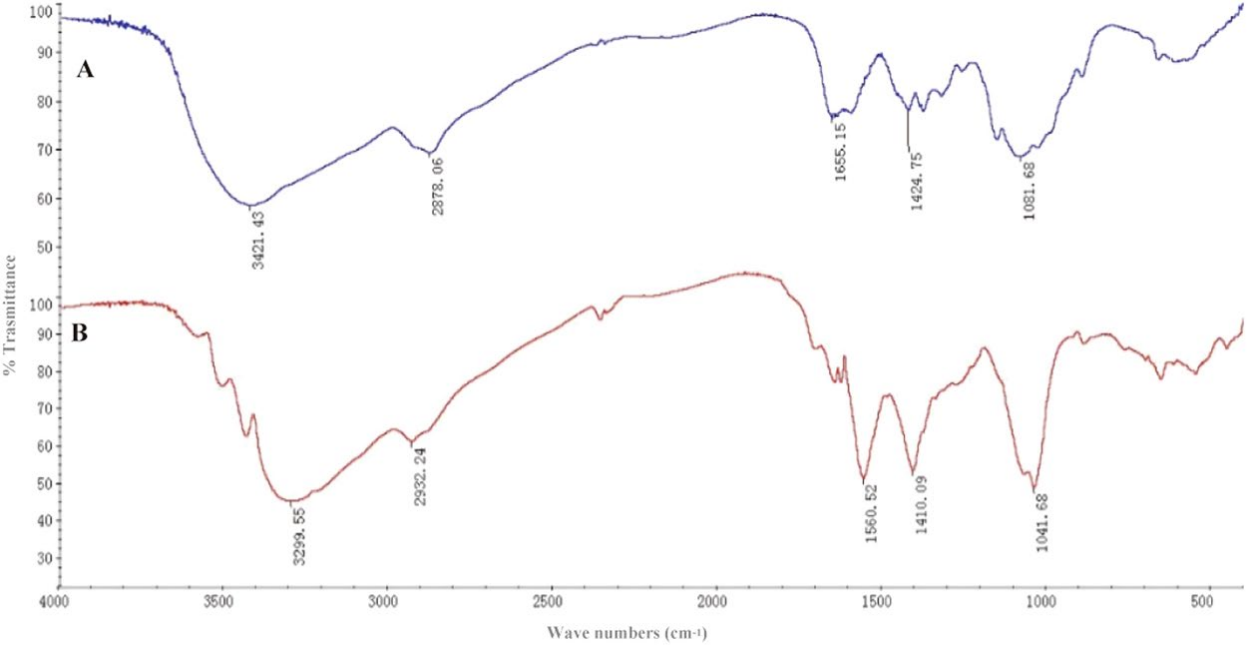
FTIR spectra of chitosan (**A**) and GC (**B**).

Following hydrolysis of chitosan using bi-enzyme single-step technique, the product (hydrolysate; GC) was characterized by HPLC, FTIR, and chemical analyses as described in “Methods”. Except for the mobile solvent peak (around 3 min), the HPLC spectrum displayed a main peak at 6.027 min (Fig. 1B), identical to that of standard D-GlcN hydrochloride (Fig. 1A). GC also showed several small peaks (Fig. 1B). Thus, GC appeared to consist of GlcN plus other minor chitosan derivatives. FTIR spectra of chitosan and GC (Fig. 2) showed similar peaks at 3299–3421 cm^−1^ (-OH and -NH2), 2878–2932 cm^−1^ (C-H stretching), 1556–1652 cm^−1^ (C=O stretching), 1410–1424 cm^−1^ (asymmetrical C-H bending of CH_2_ group), and 1041–1081 cm^−1^ (C-O-C)^4,18^. These findings indicate strong similarity between the basic structures of chitosan and GC.

Other properties of GC are summarized in Table 1. GC was composed mainly of GlcN (80.00%) and ChOS (9.80%), consistent with HPLC findings. Smaller amounts of water (8.36%) and ash (1.36%) were present. No microbes were detected. Concentrations of heavy metals were Hg 0.0022 mg/kg, Cd 0.0081 mg/kg, Pb 0.69 mg/kg, and As 0.491 mg/kg. The above measurements conform to the Chinese national standards (Guobiao) issued by the SAC for chitosan as applied to food (GB.16740–2014).

**Table 1:** Properties of GC.

Property	Value
Glucosamine content (%)	80.00
Chitooligosaccharides content (%)	9.80
Water content (%)	8.36
Ash content (%)	1.36
Hg content (mg/Kg)	0.0022
Cd content (mg/Kg)	0.0081
Pb content (mg/Kg)	0.6900
As content (mg/Kg)	0.4910
Total number of colony (cfu/g)	ND

ND: not detected.

### Effect of GC on knee joint swelling in mice

In the mouse OA model, knee cartilage was damaged as described in Sec. “Methods”, resulting in joint swelling. Mean knee joint diameter at day 15 in Group II (Model group) was significantly higher (*p* < 0.01) than in Group I (NC) (Table 2). Joint diameters from day 6 to 15 in positive control (GlcN sulfate) group (III) and three GC groups (IV, V, VI) showed significant (*p* < 0.05 or < 0.01) decreases in comparison with Model group. Thus, GC caused notable relief of knee swelling in the OA model.

**Table 2:** Knee joint diameters of mice in different groups during 15 d.

Day	NC	Model	Positive control	GC		
				40 mg/kg·bw·d	80 mg/kg·bw·d	160 mg/kg·bw·d
0d	5.86 ± 0.06^##^	8.08 ± 0.13^**^	8.12 ± 0.16^**^	8.27 ± 0.10^**^	8.05 ± 0.11^**^	8.24 ± 0.14^**^
3d	6.34 ± 0.05^##^	8.03 ± 0.13^**^	8.16 ± 0.10^**^	8.02 ± 0.13^**^	8.00 ± 0.10^**^	7.91 ± 0.10^**^
6d	6.30 ± 0.07^##^	8.15 ± 0.13^**^	7.77 ± 0.09^**,#^	7.88 ± 0.12^**^	7.69 ± 0.16^**,##^	7.64 ± 0.09^**,##^
9d	6.32 ± 0.06^##^	8.01 ± 0.22^**^	7.87 ± 0.09^**^	7.60 ± 0.12^**,#^	7.60 ± 0.12^**,#^	7.58 ± 0.11^**,#^
12d	6.22 ± 0.07^##^	7.88 ± 0.14^**^	7.48 ± 0.12^**,#^	7.49 ± 0.10^**,#^	7.69 ± 0.10^**^	7.32 ± 0.08^**,##^
15d	6.29 ± 0.04^##^	7.98 ± 0.10^**^	7.47 ± 0.09^**,##^	7.32 ± 0.07^**,##^	7.45 ± 0.08^**,##^	7.29 ± 0.07^**,##^

NC: normal control (Group I). Model: Group II. Positive control: Group III. GC 40/80/160: Groups IV, V, VI. ^*^p < 0.05 for comparison to NC. ^**^p < 0.01 for comparison to NC. ^#^*p* < 0.05 for comparison to Model group. ^##^*p* < 0.01 for comparison to Model group.

### Effect of GC on immune organ indices

**Figure 3:**
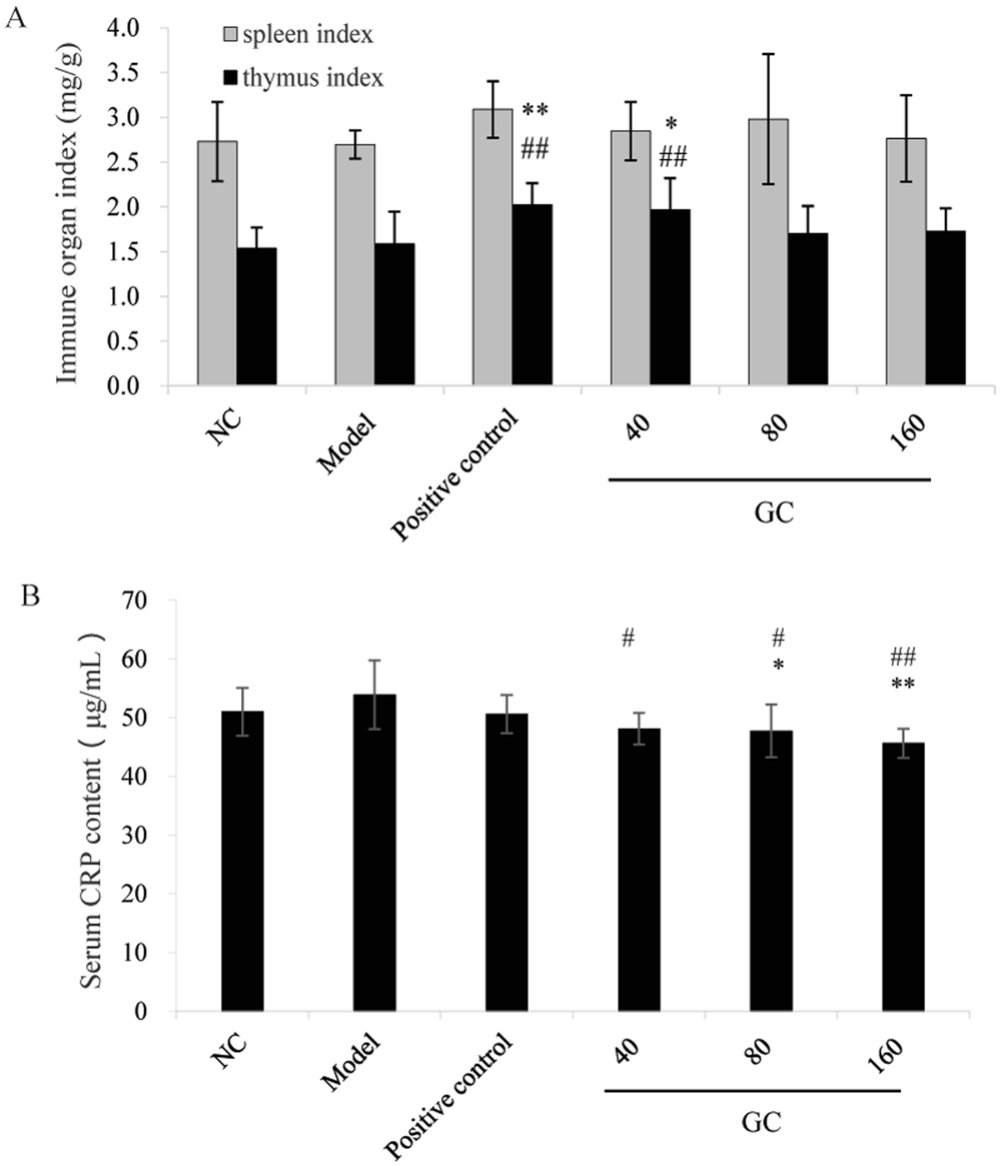
Effect of GC on immune organ indices and serum levels of C-reactive protein (CRP) for Groups I to VI. NC: normal control (Group I). Model: Group II. Positive control: Group III. GC 40/80/160: Groups IV, V, VI. ^*^p < 0.05 for comparison to NC. ^**^p < 0.01 for comparison to NC. ^#^*p* < 0.05 for comparison to Model group. ^##^*p* < 0.01 for comparison to Model group.

Spleen index was higher for the GC groups and positive control group than for Model group, but the differences were not significant (Fig. 3A). Thymus index was significantly higher for lowest-dosage GC group (*p* < 0.05) and positive control group (*p* < 0.01) than for Model group. These findings indicate that GC has immunomodulatory properties.

### Effect of GC on serum levels of CRP

CRP expression typically increases as a function of inflammation. Serum CRP levels in the three GC groups (IV, V, VI) decreased significantly (*p* < 0.05, <0.05, <0.01, respectively) and dose-dependently in comparison with Model group (Fig. 3B). CRP level of positive control group did not differ significantly from that of Model group. These findings indicate that GC can regulate CRP expression in mouse sera.

### Effect of GC on serum levels of four cytokines

**Figure 4:**
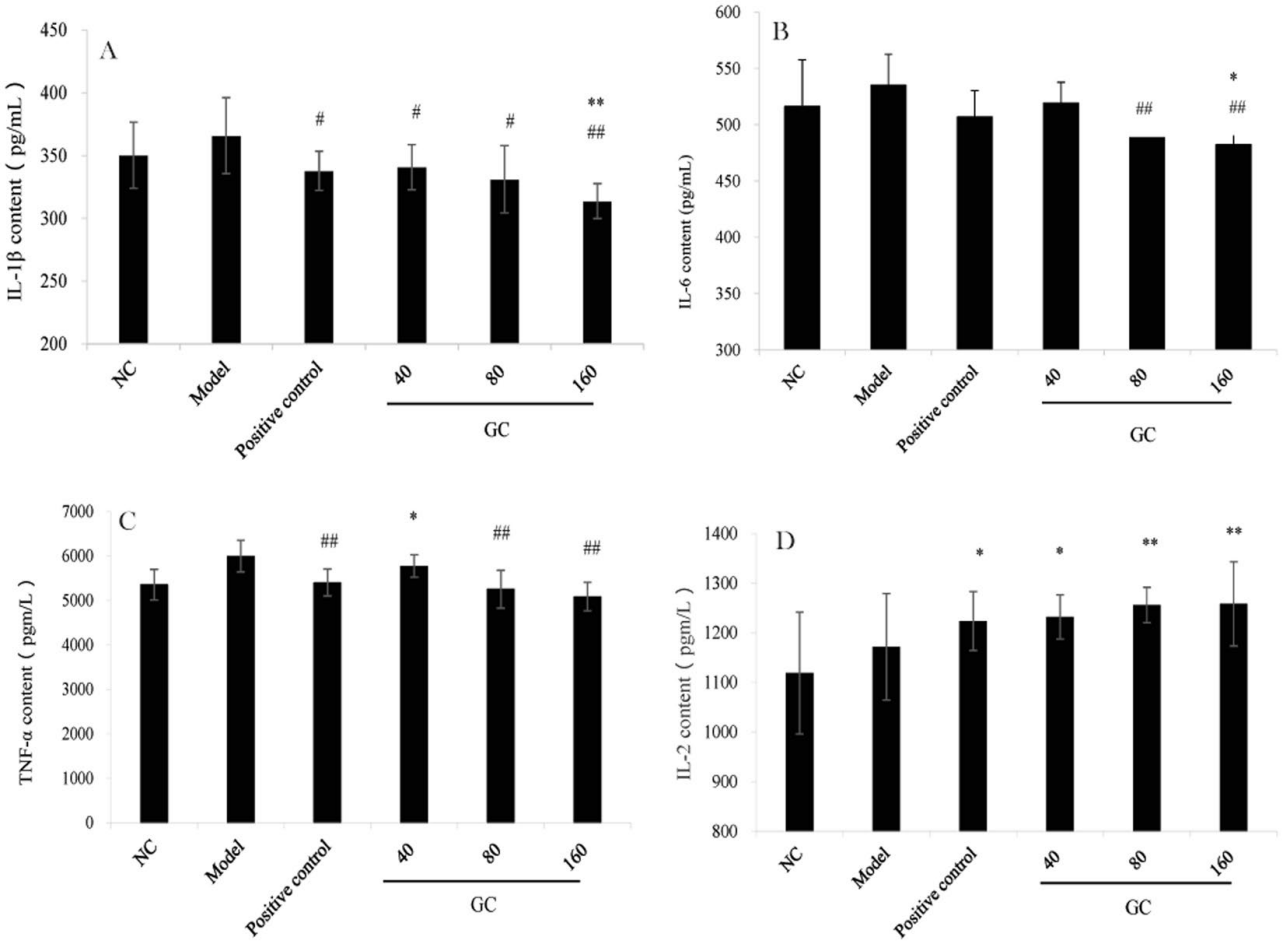
Effect of GC on serum cytokine levels. (**A**) IL-1β. (**B**) IL-6. (**C**) TNF-α. D: IL-2. Notations as in Fig. 3.

Inflammatory responses in mice are generally related to altered levels of serum cytokines. We examined levels of three pro-inflammatory cytokines (IL-1β, IL-6, TNF-α) and one anti-inflammatory cytokine (IL-2). Levels of the pro-inflammatory cytokines were higher in Model group than in NC group (Fig. 4A,B,C), indicating the occurrence of inflammatory phenomena in the model. IL-1β levels in the three GC groups were significantly (*p* < 0.05, *p* < 0.05, and *p* < 0.01) and dose-dependently lower than in Model group (Fig. 4A). IL-6 and TNF-α levels were significantly (*p* < 0.01) lower in Groups V and VI than in Model group (Fig. 4B,C). Levels of the three pro-inflammatory cytokines in the highest-dosage GC group were lower than those for positive control group. Levels of anti-inflammatory cytokine IL-2 in all three GC groups increased significantly (*p* < 0.01) and dose-dependently in comparison with NC group (Fig. 4D).

### Effect of GC on serum immunoglobulin levels

**Figure 5:**
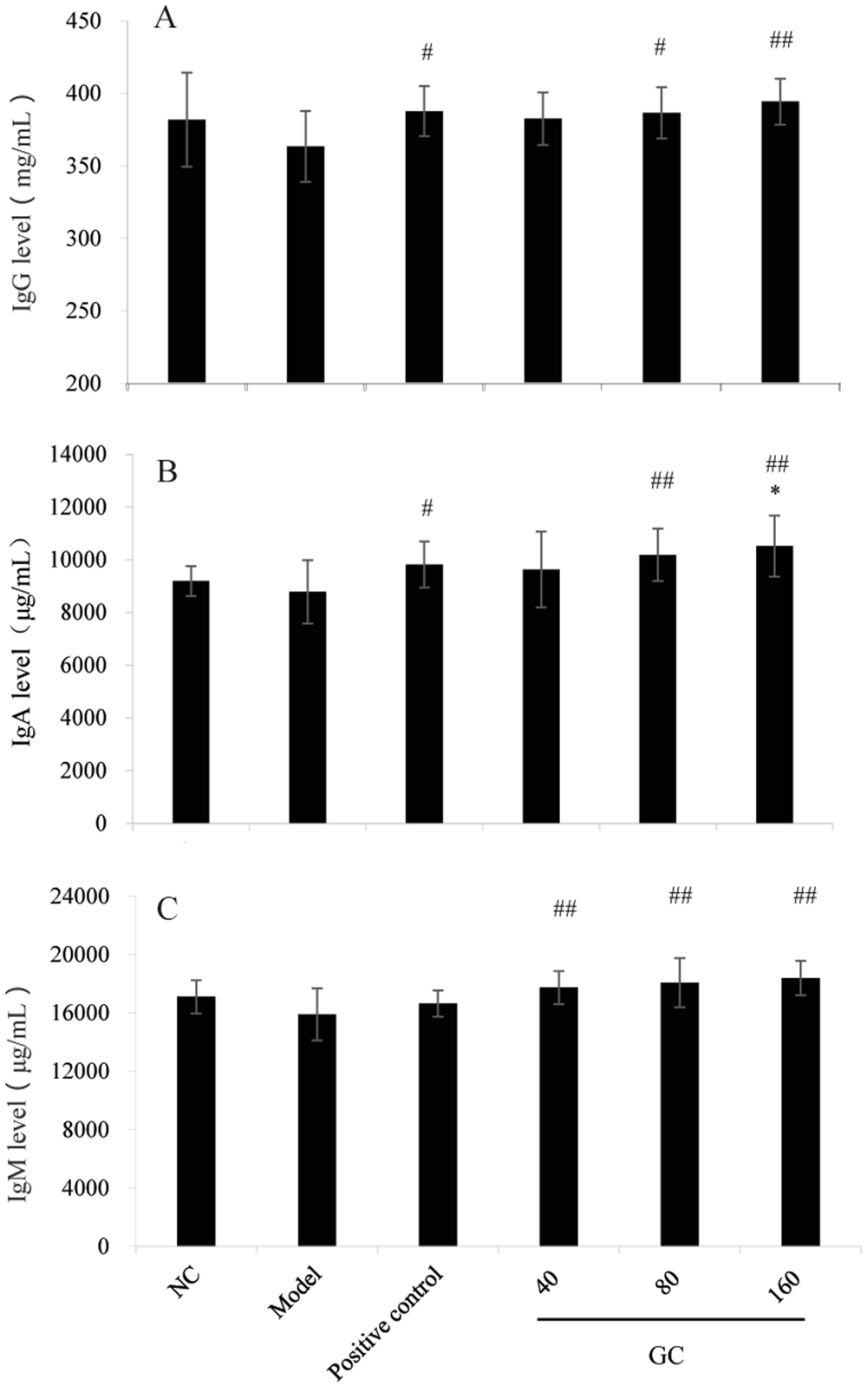
Effect of GC on serum immunoglobulin levels. (**A**) IgG. (**B**) IgA. (**C**) IgM. Notations as in Fig. 3.

We measured serum levels of the three types of immunoglobulin (IgG, IgA, IgM) in the six groups. IgG and IgA levels in Groups V and VI, and positive control group, were significantly (*p* < 0.05 or <0.01) higher than in Model group (Fig. 5A,B). IgA levels in Groups V and VI were also significantly higher than in NC group (Fig. 5B). IgM level of Model group was highly significantly (*p* < 0.01) lower than in the three GC groups, but did not differ significantly from that of positive control group (Fig. 5B,C).

### Effect of GC on repair of knee joint damage

**Figure 6:**
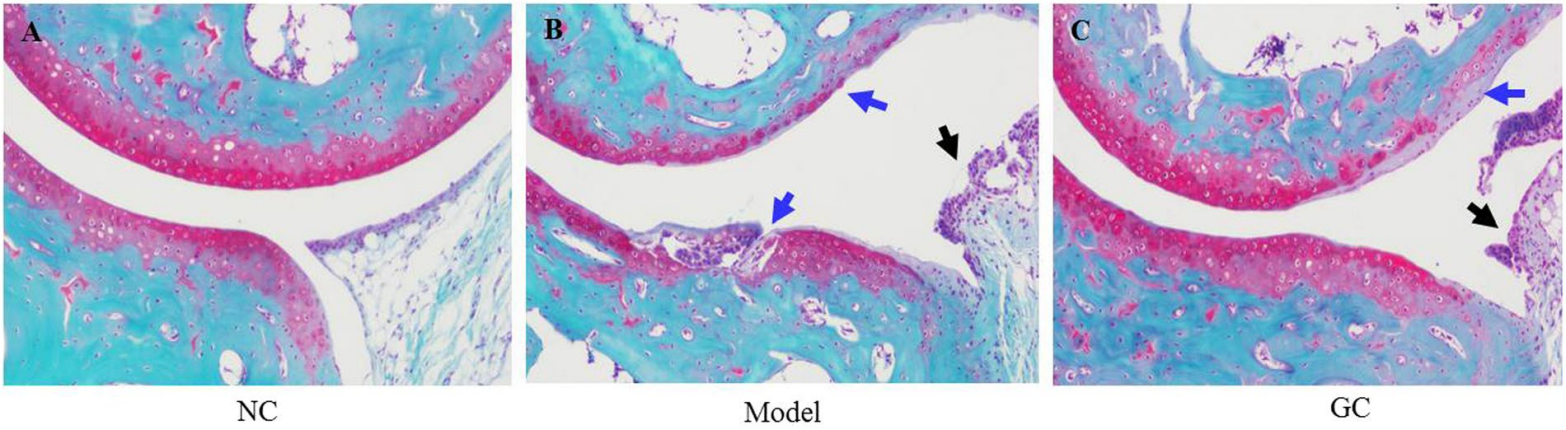
Safranin O-fast green staining of knee joint in NC, Model, and highest-dosage GC groups (Groups I, II, VI). Black arrows: synoial hyperplasia. Blue arrows: cartilage damage.

**Figure 7:**
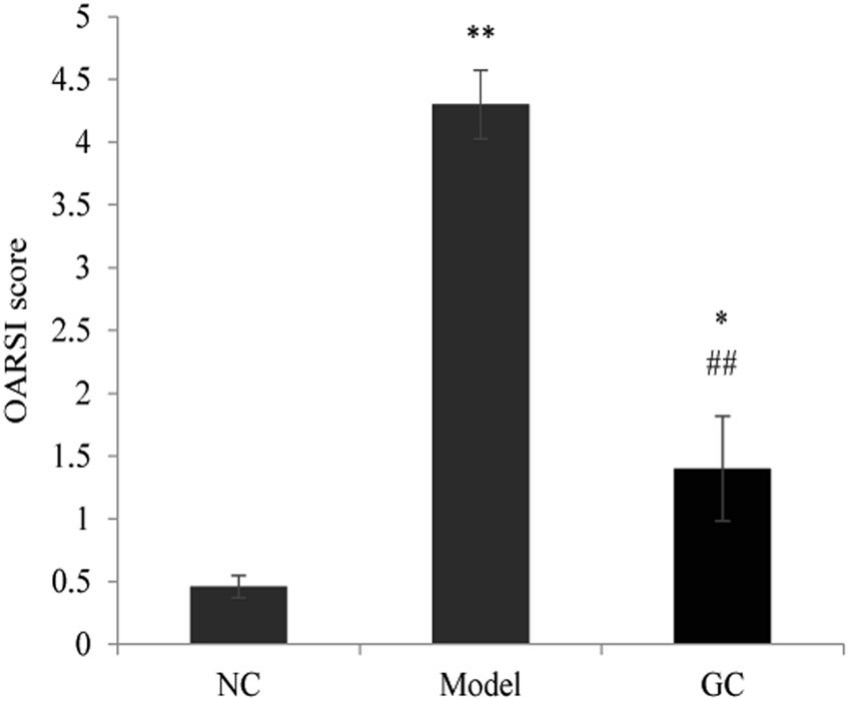
OARSI scores of the knee joint in NC, Model and highest-dosage GC groups. Notations as in Fig. 3.

Tissues around knee joints (cartilage, meniscus, synovial membrane) were damaged in the OA model groups. Knee joints of NC, Model, and highest-dosage GC groups were examined by safranin O-fast green staining at the end of the experimental period. In NC group, meniscus was intact, cartilage was smooth, and tidemark was continuous (Fig. 6A). In contrast, Model group showed serious damage: thinner and broken cartilage (blue arrow)synovial hyperplasia (black arrow) and discontinuous tidemark (Fig. 6B). Highest-dosage GC group (Group VI) showed much lower degree of knee joint damage. Broken cartilage was repaired obviously, and the tidemark was mainly integrated (Fig. 6C). According to the Osteoarthritis Research Society International (OARSI) histological assessment method of OA in the mouse^19^, the above-mentioned three groups were scored (Fig. 7). The score of Model group was significantly higher than that of NC group (*p* < 0.01). However, GC treatment for 15 d significantly decreased the score (*p* < 0.05, in comparison with Model group), indicating that GC contributes to repair of damage in OA model mice.

## Discussion

Glucosamine (GlcN), a naturally occurring monosaccharide with attached amino group, has been shown in numerous studies to play major roles in cartilage formation and repair, and has been widely used in preparation of nutritional products, functional foods, and medicines. It is also used industrially in formulation of GlcN salts (GlcN sulfate [“GS”] and GlcN hydrochloride [“GH”]) by chemical hydrolysis. However, because the terms “GS” and “GH” are ambiguous, and have been used to refer to various chemical substances^14^, there has been much controversy and disagreement regarding therapeutic evaluation. When a GlcN salt enters the human stomach, it dissociates completely to GlcN. Laverty, *et al*.^20^ detected free GlcN in synovial fluid of horses, in which its concentration remained elevated even 12 h after dosing. There have been many attempts to prepare free GlcN by hydrolysis of chitosan using specific enzymes such as chitosanase and exo-β-D-glucosaminidase^21–24^. However, industrial application of these enzymes for free GlcN production is limited by their low activity. In previous studies, we produced recombinant chitosanase (McChoA) and exo-β-D-glucosaminidase (AorCsxA), and demonstrated their high enzymatic activities following fermentation at high cell density^25,26^. McChoA and AorCsxA, by themselves, produce ChOS and free GlcN respectively. In the present study, we used McChoA and AorCsxA in combination (bi-enzyme single-step hydrolysis) to produce a mixture of GlcN (80.00%) and ChOS (9.80%), termed GC. In view of healthcare budget constraints, it is increasingly important to evaluate the cost-effectiveness of therapeutic treatments. Because of the high enzymatic activities of McChoA and AorCsxA, GC production cost was not much higher than that of previous chemical methods, and product components were clearly identifiable. Thus, our novel technique has strong potential for industrial production of GC.

Qian, *et al*.^17^ reported that chitosan was an effective absorption enhancer for GlcN. ChOS, a smaller, highly soluble polymer of GlcN, may also promote GlcN bioactivity. de Assis, *et al*.^16^ found that a small amount of ChOS blocked the cytotoxic effect of GlcN against mouse embryonic fibroblast cell line 3T3. ChOS comprise a series of active compounds that display antitumor, antioxidant, and anti-inflammatory properties. During the pathogenesis and progression of OA, patients typically undergo oxidative stress. ChOS exerts antioxidant activity *in vitro* and *in vivo*^27–31^, and therefore may be useful in treatment of OA when applied in combination with GlcN. ChOS also display anti-inflammatory effects^8,32^, and undergo high-affinity binding with the chitinase-like proteins YKL-40 and YKL-39, which are involved in a variety of physiological processes (*e.g*., tissue remodeling, chondrocyte repair, inflammation) and serve as specific biomarkers for OA progression^33,34^. These observations led us to develop the novel GC preparation technique described here.

OA progression is associated with increased levels of inflammatory mediators^15^. Induction of inflammatory mediator synthesis in OA tissues depends on various cytokines, particularly IL-1β and TNF-α^35^. Kuyinu, *et al*.^9^ recently described an inflammatory mechanism for the initial stages of OA, and proposed cytokines and inflammatory mediators as potential therapeutic targets. In a study by Man and Mologhianu^36^, OA development was triggered by release of cytokines (IL-1, IL-6, TNF-α). In the present study, IL-1β, IL-6, and TNF-α levels were significantly and dose-dependently inhibited by GC, in agreement with previous reports. In previous studies, GlcN downregulated expression of TNF-α gene via O-N-acetyl-GlcN modification of Sp1^37^, and inhibited IL-1β-induced production of IL-8, nitric oxide, and PGE2 by synovial cells^7^. In contrast, level of anti-inflammatory cytokine IL-2 was significantly and dose-dependently enhanced by GC in the present study. Inflammation is a normal, physiological immune system response against pathogens, toxic chemicals, or physical injury^11^. In this study, GC administration led to increases of thymus index and IgG, IgA, and IgM levels. Thus, GC evidently plays a key role in inhibition of inflammation, in addition to its involvement in joint tissue remodeling.

The pathological effects of OA may include pain, stiffness, and swelling of joints. In our OA mouse model, knee swelling symptoms were significantly relieved following GC treatment. OA progression often involves degenerative changes in the meniscus, articular cartilage, and synovial membrane. In this study, meniscus damage, cartilage damage, and synovial hyperplasia in the knee joint were partially ameliorated in the GC-treated groups. Our findings indicate that GC has strong potential as a therapeutic agent against OA and other inflammatory diseases. Optimal proportions of GlcN and ChOS in GC, for maximal therapeutic effect, are being investigated in our ongoing studies.

## Methods

### Preparation of hydrolyzed GlcN + ChOS (“GC”)

Recombinant chitosanase (termed McChoA; 1100.2 U/mL) and exo-β-D-glucosaminidase (termed AorCsxA; 222 U/mL) from *Pichia pastoris* were produced in our laboratory^25,26^. McChoA and AorCsxA were used together to produce a targeted sample by single-step hydrolysis. Chitosan (MW 20–30 KDa, degree of deacetylation 85–95%) and acetate-NaAc buffer (pH 5.5) at room temperature were mixed and stirred until dissolved. McChoA (20 U/g) and AorCsxA (35 U/g) were added to the chitosan solution (final concentration 10%) and the resulting reaction solution was incubated at 50 °C for 10 h. The targeted sample was obtained by lyophilization. The hydrolysate, GlcN + ChOS, is referred to as “GC” hereafter, for convenience.

### Characterization of GC

GC was analyzed by Fourier transform infrared spectroscopy (FTIR) and high-performance liquid chromatography (HPLC). FTIR spectra of original chitosan and GC were collected in KBr pellets on a Nicolet Nexus FTIR 470 spectrophotometer (Thermo Nicolet; Madison, WI, USA) at wavelength range 400–4000 cm^−1^. HPLC was performed using a LC-20AT system (Shimadzu Corp.; Kyoto, Japan). D-GlcN hydrochloride standard (from Sigma-Aldrich; St. Louis, MO, USA) and GC solutions were loaded separately on a Luna NH2 100 A column (250 × 4.60 mm, 5 µm) (Phenomenex Inc.; Torrance, CA, USA), eluted with acetonitrile/water (ratio 3:1) at flow rate 1 mL/min, and retention time was recorded. A calibration curve was obtained by plotting concentrations of D-GlcN hydrochloride standard solutions vs. retention time, and used for determination of GlcN content in GC. Carbohydrate content of ChOS was determined by the method of Miller^38^. Water content was measured by the direct dry method of the State Standard of the P.R.C. (SAC) (GB5009.3-2010). Ash content and total colony number were measured respectively by SAC methods GB5009.4-2010 and GB4789.2-2010. Heavy metal content was determined by the method of Bortey-Sam, *et al*.^39^.

### Animal experiments

C57BL/6 mice (male; 20 ± 2 g) from the Animal Experimental Center, Hubei Center for Disease Control, China were maintained on a 12 h dark/12 h light cycle at 20 °C with ad lib access to standard laboratory food pellets and water, and allowed to adapt to the environment for 1 week. All procedures involving animals and their care were approved by the animal ethics committee of Renmin Hospital of Wuhan University (Approval number: 2013127, EXP#S01314040T), Wuhan, Hubei, China. All animal experiments complied with the Guide for the Care and Use of Laboratory Animals by the National Institutes of Health. Animals were divided randomly into 6 groups with 10 mice per group. Group I was the normal control (hereafter termed “NC”). Animals of Groups II to VI, comprising the experimental OA model, were sedated with 10% chloral hydrate, and cartilage of the knee meniscus was surgically damaged by the method of Chen, *et al*.^40^. The 6 groups were then administered various solutions intragastrically for a 15-day experimental period. Groups I (NC) and II (hereafter termed “Model”) received normal saline solution (0.9%, w/v) orally. Group III (positive control) received Swisse GlcN sulfate (from Swisse Wellness Pty; Melbourne, Australia) at a dosage of 80 mg/kg body weight per day (mg/kg·bw·d). Groups IV, V, and VI received GC at low, intermediate, and high dosages (40, 80, and 160 mg/kg·bw·d respectively). Knee diameters were measured at 4-day intervals using a Vernier caliper. Animals were weighed and killed by cervical dislocation 24 h after final drug administration.

### Immune organ indices

Within each group, spleen and thymus were aseptically removed and weighed, and immune organ indices were calculated as:

Spleen index = mean spleen weight of group/mean body weight of group.

Thymus index = mean thymus weight of group/mean body weight of group.

### Biochemical assays

For each animal, a blood sample was taken from the orbital sinus and centrifuged at 3000 × *g* for 10 min at 4 °C to obtain serum. Levels of cytokines (IL-1β, IL-2, IL-6, TNF-α) in serum were analyzed using ELISA assay kits (Nanjing Jiancheng Bioengineering Institute; Nanjing, China). C-reactive protein (CRP) concentration and antibody (IgA, IgM, IgG) levels in serum were determined using corresponding assay kits from the same manufacturer.

### Histopathology analysis

For each animal, knee joint biopsy was performed 24 h after final drug administration. Undamaged distal parts of bilateral knee joint femur (1 cm) were excised using bone shears. Surrounding soft tissues were removed, and knee joints were immediately fixed in neutral buffered formalin, stained with safranin O-fast green, and sent out for histopathological examination (Servicebio Co.; Wuhan, China).

### Statistical analysis

Data were expressed as mean ± SD. Means were compared by Student’s t-test using the SPSS for Windows software program, v. 19.0 (SPSS Inc.; Chicago, IL, USA). Differences with *p* < 0.05 and *p* < 0.01 were considered significant and highly significant, respectively.
